# Supplementary material for: Globular Aggregates Stemming from the Self-Assembly of an Amphiphilic N-Annulated Perylene Bisimide in Aqueous Media
Source: Nanomaterials (Basel). 2021 May 31;11(6):1457. doi: 10.3390/nano11061457 (PMC8228590; doi:10.3390/nano11061457)
Supplement: Supplementary file 1 [file nanomaterials-11-01457-s001.zip › nanomaterials-1220570-supplementary.pdf]

Supplementary Materials

Globular Aggregates Stemming from the Self-Assembly of an Amphiphilic *N*-Annulated Perylene Bisimide in Aqueous Media

Manuel A. Martínez [1,†], Elisa E. Greciano [1,†], Jorge Cuéllar [2], José M. Valpuesta [2] and Luis Sánchez [1,*]

[1] Departamento de Química Orgánica, Facultad de Ciencias Químicas, Universidad Complutense de Madrid, 28040 Madrid, Spain; manuma08@ucm.es (M.A.M.); eegreciano@ucm.es (E.E.G.)
[2] Centro Nacional de Biotecnología, Campus de la Universidad Autónoma de Madrid, 28049 Madrid, Spain; jcuellar@cnb.csic.es (J.C.); jmv@cnb.csic.es (J.M.V.)
* Correspondence: lusamar@ucm.es
† These authors contributed equally to this work.

1. Synthetic details and characterization 2

2. Collection of spectra 4

1. Synthetic details and characterization

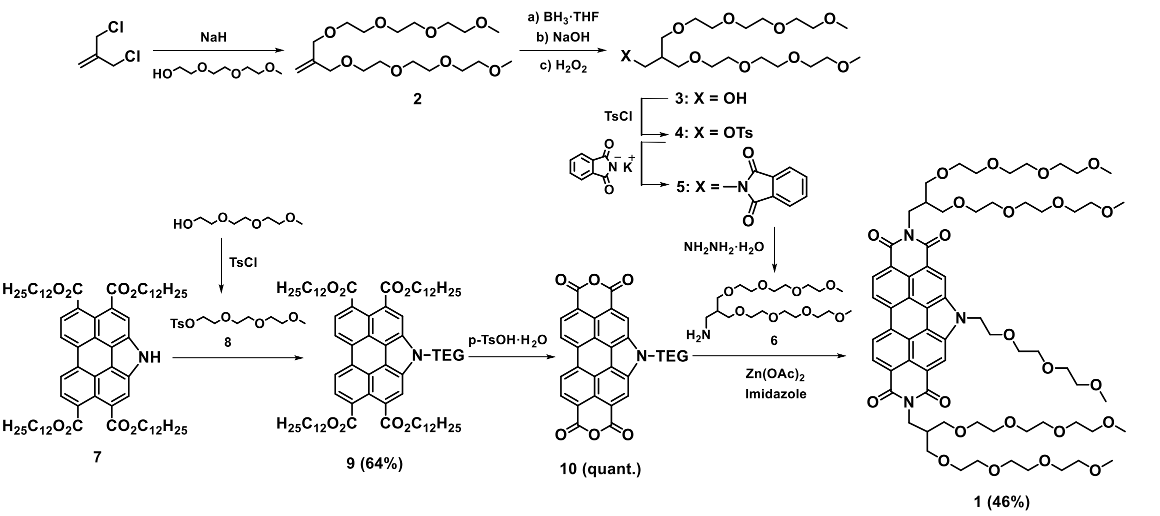

Scheme S1. Synthesis of the amphiphilic *N*-annulated PBI **1**.

Compounds **2**[S1, S2] and **6-10**[S3] were prepared according to previously reported synthetic procedures and showed identical spectroscopic properties to those reported therein.

[S1] Gupta, R. K.; Pathak, S. K.; Pradhan, B.; Rao, D. S. S.; Prasad, S. K.; Achalkumar, A. S. Self-assembly of luminescent N-annulated perylene tetraesters into fluid columnar phases. *Soft Matter* **2015**, *11*, 3629–3636.

[S2] Gupta, R. K.; Rao, D. S. S.; Prasad, S. K.; Achalkumar, A. S. Columnar Self-Assembly of Electron-Deficient Dendronized Bay-Annulated Perylene Bisimides. *Chem. Eur. J.* **2018**, *24*, 3566–3575.

[S3] Buendía, J.; García, F.; Yélamos, B.; Sánchez, L. Transfer and amplification of chirality in Phe-based C3-symmetric non-ionic amphiphiles. *Chem. Comm.* **2016**, *52*, 8830-8833.

Tetradodecyl 1-(2-(2-(2-methoxyethoxy)ethoxy)ethyl)-1*H*-phenanthro[1,10,9,8-*cdefg*]carbazole-3,4,9,10-tetracarboxylate (9)

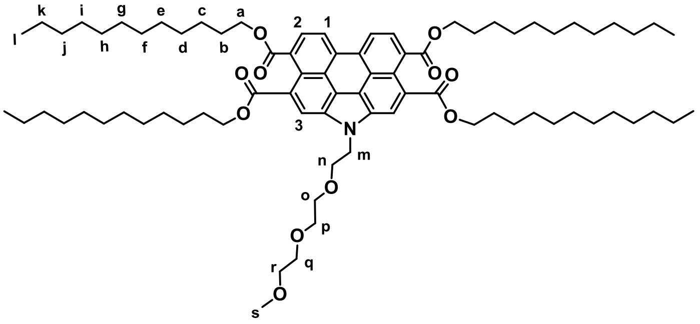

$C_{79}H_{121}NO_{11}$
Exact Mass: 1259.894
Mol. Wt.: 1260.8061

NaH (25 mg, 0.61 mmol) was slowly added to a mixture of compound **7** (500 mg, 0.45 mmol) and compound **8** (200 mg, 0.61 mmol) in dry THF (11 mL). The resulting mixture was refluxed overnight under argon atmosphere. After that, water (3 mL) was slowly added and the resulting mixture stirred at 0 ºC for 30 min. After evaporation of the solvent under reduced pressure, the residue was extracted with diethyl ether and washed three times with water. The resulting organic layer was dried over $MgSO_4$ and the solvent was evaporated under reduced pressure. The obtained residue was precipitated and washed with methanol to afford compound **9** as an orange solid (360 mg, 64 %). ^{1}H NMR ($CDCl_3$, 300 MHz) δ 8.46 (2H, H_1, d, *J*=8.0 Hz), 8.24 (2H, H_3, s), 8.16 (2H, H_2, d, *J*=8.0 Hz), 4.84 (2H, H_m, t, *J*=5.0 Hz), 4.42 (8H, H_a, q, *J*=6.9 Hz), 3.91 (2H, H_n, t, *J*=5.0 Hz), 3.39 (4H, H_{o+p}, m), 3.29 (2H, H_q, m), 3.15 (2H, H_r, m), 3.12 (3H, H_s, s), 1.86 (8H, H_b, quin, *J*=6.9 Hz), 1.52–1.21 (72H, H_{c-k}, br), 0.87 (12H, H_l, t, *J*=6.9 Hz); ^{13}C NMR ($CDCl_3$, 75 MHz) δ 169.6, 169.2, 132.1, 131.8, 129.1, 128.0, 127.7, 124.2, 123.3, 121.8, 118.4, 117.9, 77.4, 71.8, 71.2, 71.1, 70.7, 70.6, 65.9, 65.8, 58.9, 46.2, 32.1, 29.9, 29.8, 29.8, 29.6, 29.6, 29.5, 28.9, 26.3, 22.8, 14.3; FTIR (neat) 667, 718, 750, 802, 837, 889, 994, 1023, 1047, 1061, 1129, 1151, 1185, 1201, 1261, 1309, 1348, 1378, 1423, 1469, 1490, 1558, 1587, 1670, 1723, 2850, 2916, 2953 cm^{-1}; HRMS (MALDI-TOF) calcd. for $C_{79}H_{121}NO_{11}$ [M], 1259.8940; found, 1259.8912.

5-(2-(2-(2-methoxyethoxy)ethoxy)ethyl)-1*H*-pyrano[3',4',5':4,5]naphtho[2,1,8-*cde*]pyrano-[3',4',5':4,5]-naphtho[8,1,2-*ghi*]isoindole-1,3,7,9(5*H*)-tetraone (10)

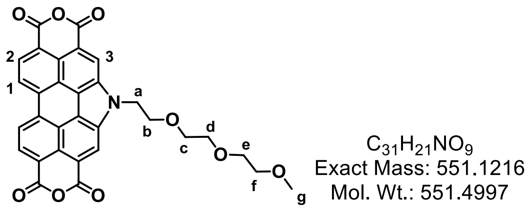

A mixture of compound **10** (0.36 g, 0.29 mmol) and *p*-toluene sulphonic acid monohydrate (0.27 g, 1.43 mmol) in toluene (5 mL) was refluxed overnight. After cooling the mixture at room temperature, the mixture was filtered and washed with methanol to afford compound **10** as a dark red solid (180 mg, quant.). FTIR (neat) 624, 699, 728, 742, 784, 800, 854, 918, 945, 1020, 1059, 1077, 1127, 1180, 1229, 1279, 1328, 1352, 1378, 1397, 1425, 1458, 1483, 1544, 1558, 1601, 1740, 1776, 2853, 2922 cm^{-1}; HRMS (MALDI-TOF) calcd. for $C_{31}H_{21}NO_9$ $[M+H]^+$, 552.1295; found, 552.1264.

2,8-bis(13-(2,5,8,11-tetraoxadodecyl)-2,5,8,11-tetraoxatetradecan-14-yl)-5-(2-(2-(2-methoxy-ethoxy)-ethoxy)ethyl)-1*H*-pyrido[3',4',5':4,5]naphtho[2,1,8-*cde*]pyrido[3',4',5':4,5]naphtho[8,1,2-*ghi*]isoindole-1,3,7,9(2*H*,5*H*,8*H*)-tetraone (1)

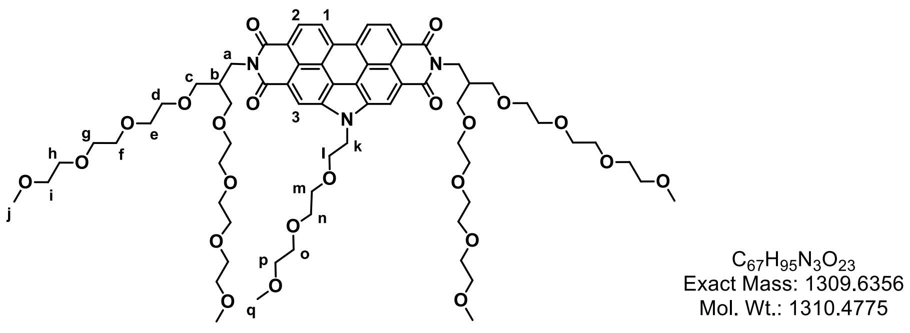

A mixture of anhydride **10** (63 mg, 0.114 mmol), amine **6** (107 mg, 0.269 mmol), zinc acetate (84 mg, 0.456 mmol) and imidazole (3 g) was subjected to a few argon/vacuum cycles and heated up at 110 ºC overnight. After cooling, the reaction mixture was extracted with chloroform and washed with HCl 1N and saturated sodium chloride solution. The organic layer was dried over $MgSO_4$ and the solvent was evaporated under reduced pressure. The resulting residue was purified by column chromatography (silica gel, chloroform/methanol 2 %) affording compound **1** as a red solid (69 mg, 46 %). ^{1}H NMR ($CDCl_3$, 300 MHz) δ 8.95 (2H, H_3, s), 8.81 (2H, H_1, d, *J*=8.0 Hz), 8.72 (2H, H_2, d, *J*=8.0 Hz), 5.04 (2H, H_k, t, *J*=4.5 Hz), 4.42 (4H, H_a, d, *J*=6.9 Hz), 4.11 (2H, H_l, t, *J*=4.5 Hz), 3.67–3.47 (64H, $H_{(c-i)+(m-p)}$, m), 3.33 (12H, H_j, s), 3.20 (3H, H_q, s), 0.85 (2H, H_b, quin, *J*=6.0 Hz), ^{13}C NMR ($CDCl_3$, 75 MHz) δ 165.3, 164.2, 135.3, 132.9, 127.7, 124.6, 123.9, 122.2, 121.8, 121.7, 119.7, 119.6, 77.6, 77.2, 72.0, 71.9, 71.1, 71.0, 70.8, 70.7, 70.6, 59.1, 59.0, 47.3, 43.3, 41.0, 38.9; FTIR (neat) 631, 741, 804, 849, 877, 936, 999, 1039, 1105, 1199, 1222, 1251, 1305, 1325, 1352, 1375, 1400, 1427, 1444, 1521, 1558, 1602, 1653, 1689, 2868 cm^{-1}; HRMS (MALDI-TOF) calcd. for $C_{67}H_{95}N_3O_{23}$ [M], 1309.6356; found, 1309.6368.

2. Collection of spectra

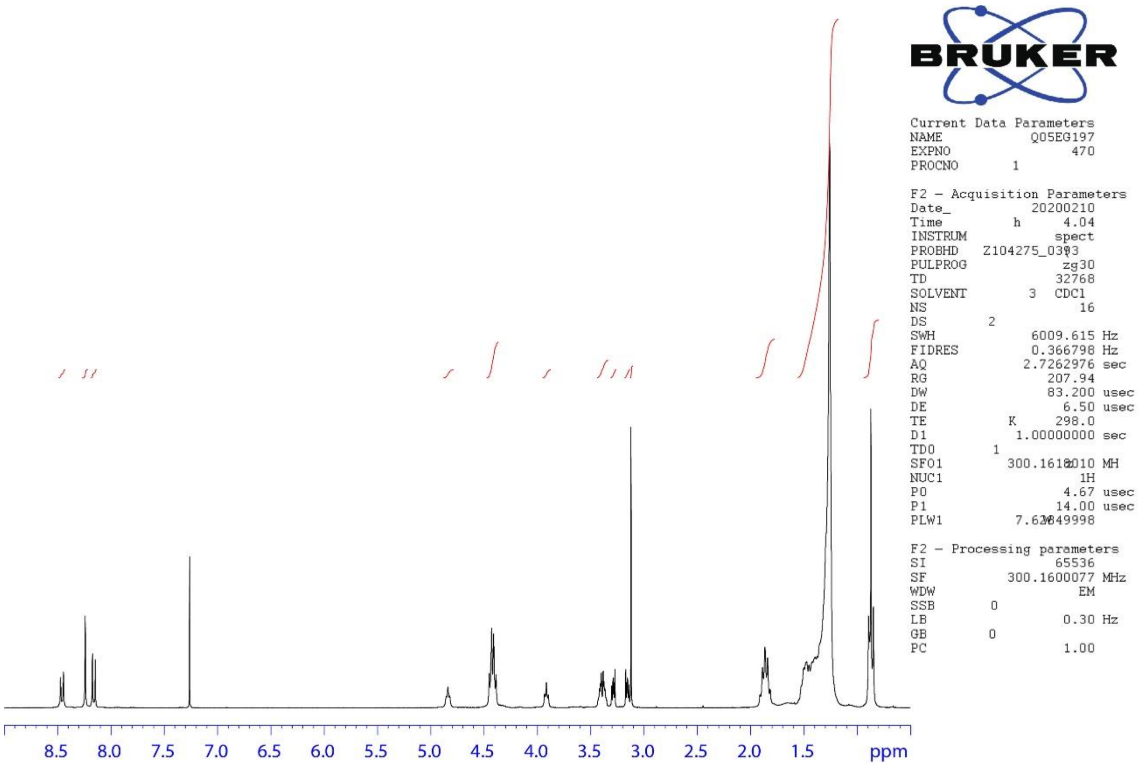

^{1}H NMR ($CDCl_3$, 300 MHz, 25 ºC) of compound **9**.

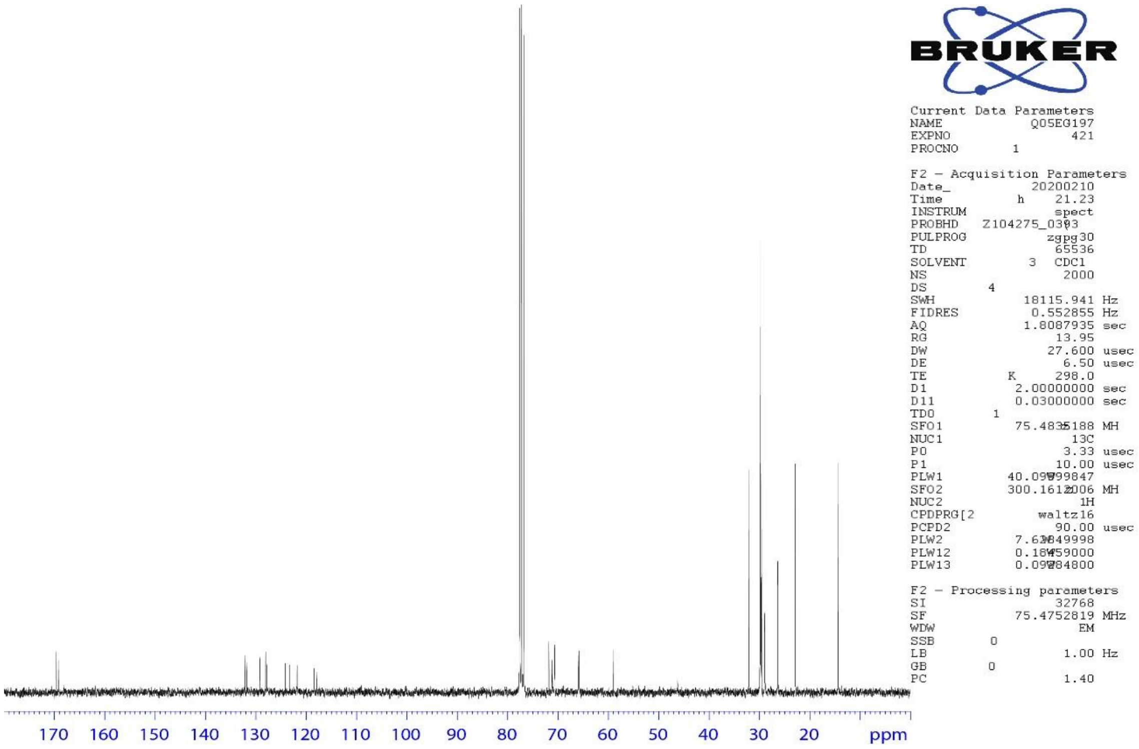

^{13}C NMR ($CDCl_3$, 75 MHz, 25 ºC) of compound **9**.

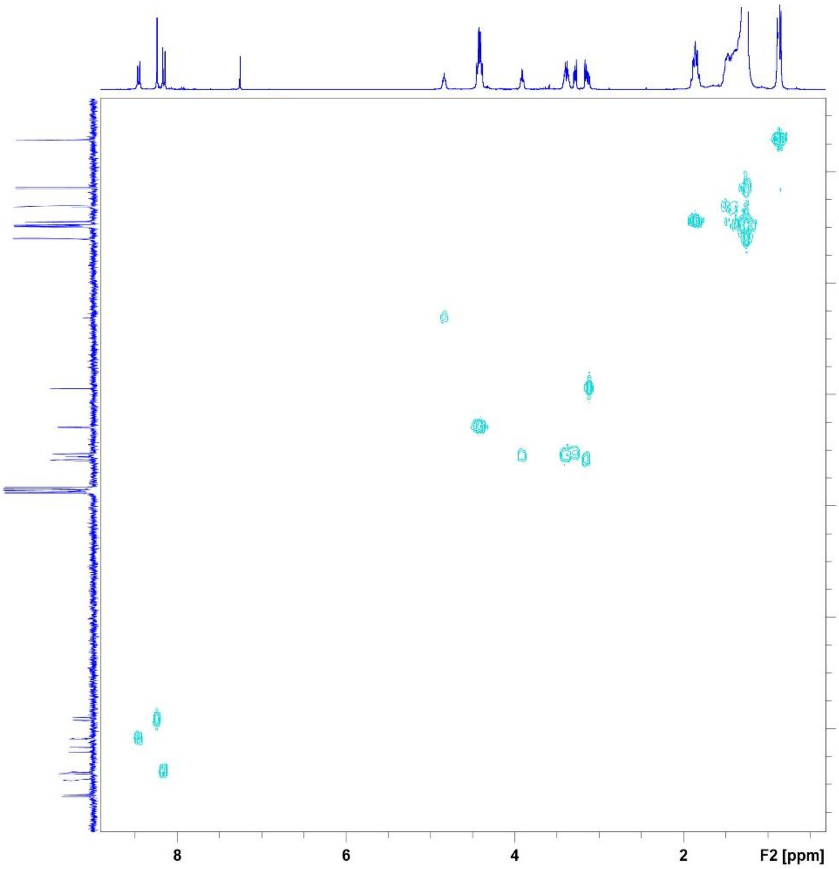

$^1H,^{13}C$-HMQCspectrum ($CDCl_3$, 25 ºC) of compound **9**.

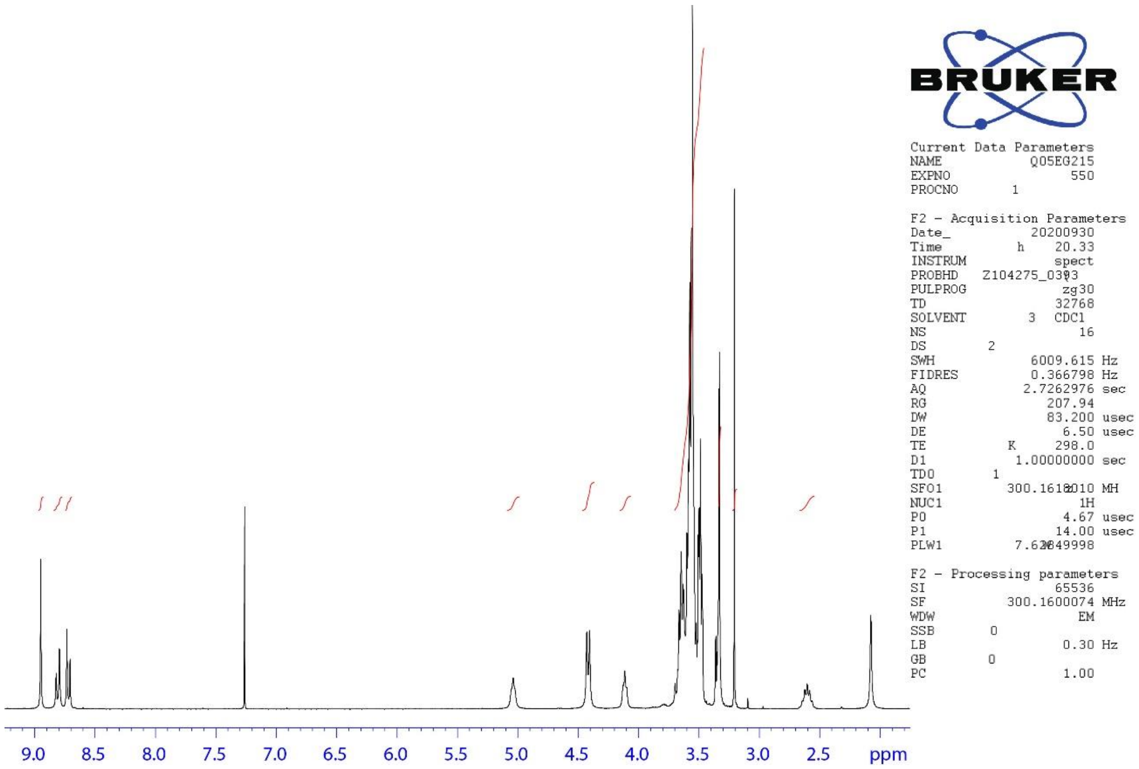

^{1}H NMR ($CDCl_3$, 300 MHz, 25 ºC) of compound **1**.

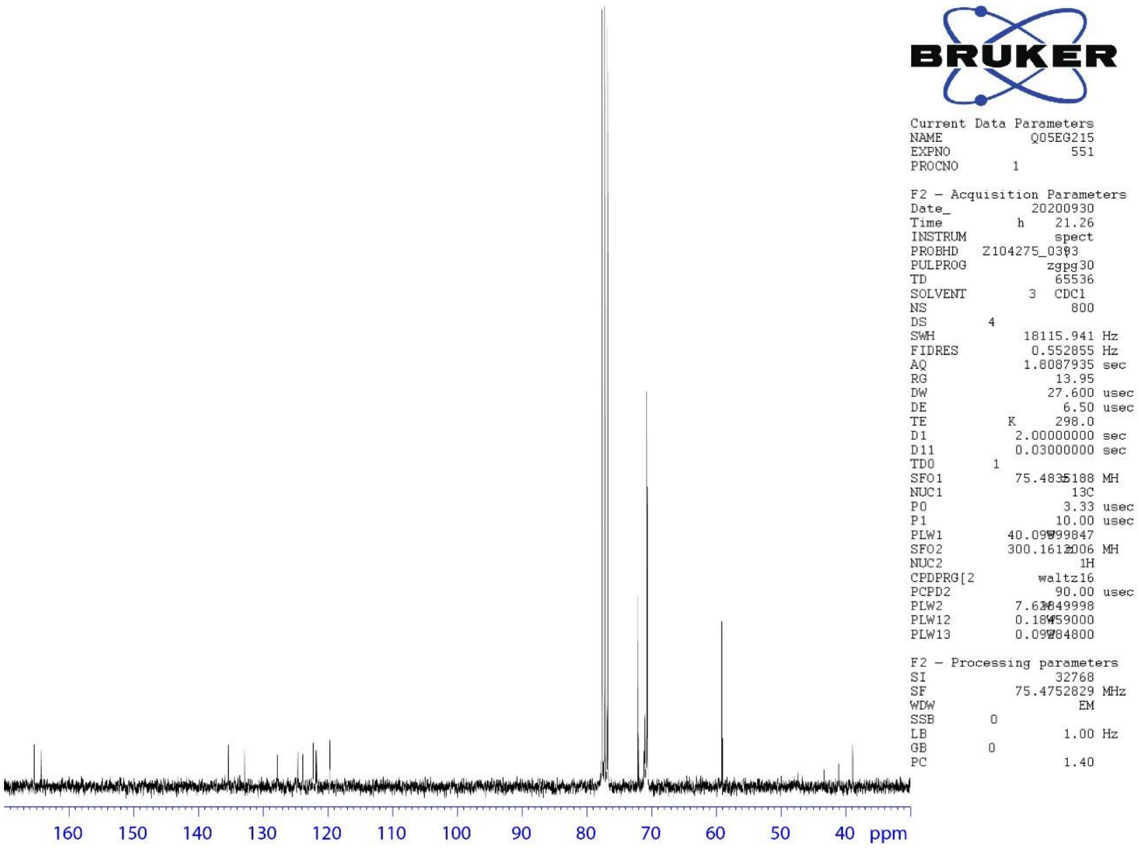

^{13}C NMR ($CDCl_3$, 75 MHz, 25 ºC) of compound **1**.

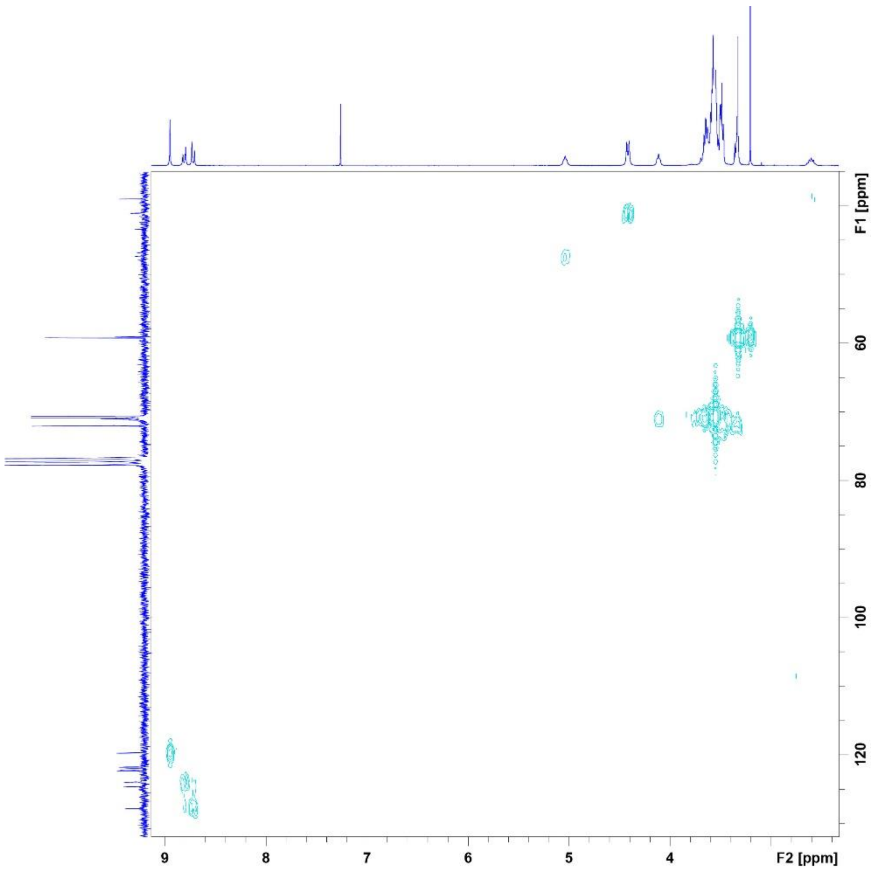

$^{1}H,^{13}C$-HMQC spectrum ($CDCl_3$, 25 ºC) of compound **1**.
